# Personal

**Published:** 1898-10

**Authors:** 


### PERSONAL.

Dr. H. G. Bentz, of Buffalo, an alumnus of Buffalo University Medical College, became a member of the Medical Society of the County of Erie, June 10, 1891. His name should have appeared in the list for that year, published in the September edition of the Journal, p. 100, in A century of medical history. It is a matter of regret that it was omitted, and we shall be glad to make corrections in any similar cases that may be brought to our notice.

Dr. Nelson W. Wilson, of Buffalo, has been appointed an acting-assistant surgeon, U. S. Army, and assigned to duty at Fort Porter, in this city. This is a most excellent appointment and reflects credit upon the Surgeon-General's office. Since the return of the Thirteenth Infantry, Dr. Wilson's duties have been of an active character, and we learn that he is giving satisfaction to the officers and the men at the fort.

Dr. Prescott Le Breton, of Buffalo, has located his office at 147 Allen street. Hours, 8-9 a. m., 1-2 and 7-8 p. m. Telephone, Tupper 2326.

